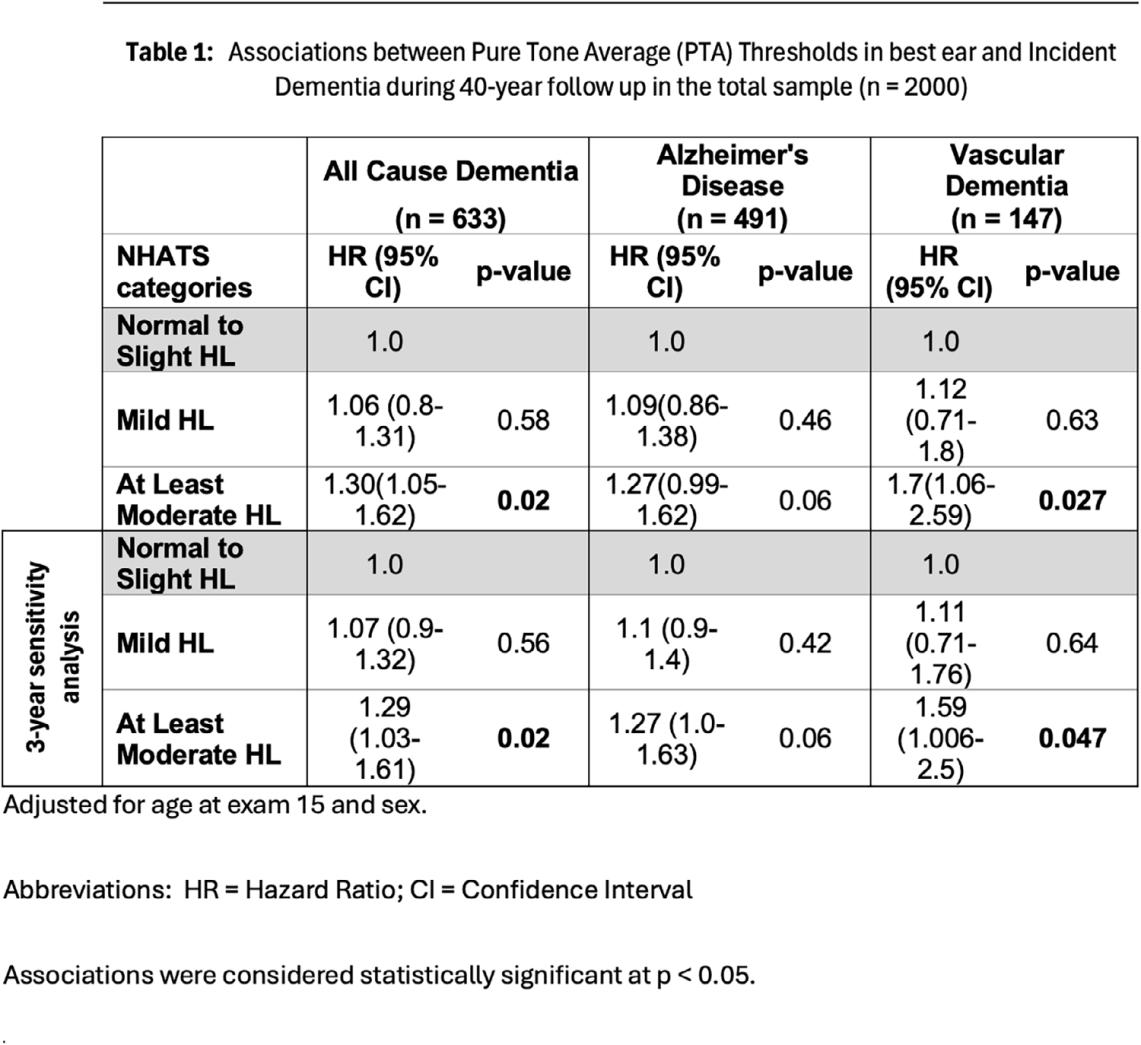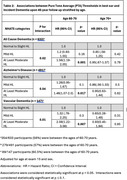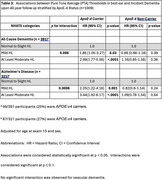# Hearing loss in mid‐life increases risk for Alzheimer's disease and vascular dementia

**DOI:** 10.1002/alz70860_105266

**Published:** 2025-12-23

**Authors:** Lily Francis, Alexa S Beiser, Sophia Lu, Sharon G Kujawa, Nancy Heard‐Costa, Francis B Kolo, Rebecca Bernal, M. Ilyas Kamboh, Bradley Duane Welling, Richard Alcabes, Monica Goss, Jayandra Jung Himali, Sudha Seshadri

**Affiliations:** ^1^ Glenn Biggs Institute for Alzheimer's & Neurodegenerative Diseases, UT Health San Antonio, San Antonio, TX, USA; ^2^ Glenn Biggs Institute for Alzheimers and other Neurodegenerative Disorders, San Antonio, TX, USA; ^3^ UT Health, San Antonio, TX, USA; ^4^ Department of Biostatistics, Boston University School of Public Health, Boston, MA, USA; ^5^ Boston University School of Public Health, Boston, MA, USA; ^6^ The Framingham Heart Study, Framingham, MA, USA; ^7^ Department of Neurology, Boston University Chobanian & Avedisian School of Medicine, Boston, MA, USA; ^8^ Boston University, Boston, MA, USA; ^9^ Harvard Medical School, Boston, MA, USA; ^10^ NHLBI Framingham Heart Study, Framingham, MA, USA; ^11^ Department of Neurology, School of Medicine, Boston University, Boston, MA, USA; ^12^ Glenn Biggs Institute, San Antonio, TX, USA; ^13^ Glenn Biggs Institute for Alzheimer's & Neurodegenerative Diseases, University of Texas Health San Antonio, San Antonio, TX, USA; ^14^ University of Pittsburgh Alzheimer's Disease Research Center (ADRC), Pittsburgh, PA, USA; ^15^ Department of Human Genetics, University of Pittsburgh, Pittsburgh, PA, USA; ^16^ City of San Antonio, San Antonio, TX, USA; ^17^ Glenn Biggs Institute for Alzheimer's & Neurodegenerative Diseases, University of Texas Health Science Center, San Antonio, TX, USA; ^18^ Department of Population Health Sciences, University of Texas Health Sciences Center, San Antonio, TX, USA; ^19^ Department of Neurology, Boston University School of Medicine, Boston, MA, USA; ^20^ Framingham Heart Study, Framingham, Boston, MA, USA; ^21^ Department of Neurology, University of Texas Health Sciences Center, San Antonio, TX, USA, San Antonio, TX, USA

## Abstract

**Background:**

Hearing loss is a risk factor for dementia, but dementia subtypes underlying this association and effect modifiers are unclear. Here we examined the association of hearing loss with all cause dementia, Alzheimer's disease and vascular dementia, and effect modifiers of this association in the Framingham Study.

**Method:**

Framingham Study participants >60 years, who underwent Pure Tone Audiometry at the 15^th^ biennial exam (1977‐1979), and had subsequent dementia surveillance for up to 40 years were included. We defined hearing loss as pure‐tone average (0.5–4.0 kHz) > 25 dB in the better ear and used National Health and Aging Trends Study (NHATS) categories: normal (range: 0 – 25dB), mild hearing loss (range: 26 – 40dB), moderate to severe hearing loss (>40dB). All cause dementia diagnosed using DSM IV criteria, Alzheimer's disease diagnosed using National Institute of Neurological and Communicative Disorders and Stroke and the Alzheimer's Disease and Related Disorders Association (NINCDS–ADRDA) criteria, vascular dementia diagnosed using the National Institute of Neurological Disorders and Stroke and the Association Internationale pour la Recherche et l’Enseignement en Neurosciences (NINDS–AIREN) NINDS‐ AIREN criteria.

**Result:**

Among 2000 participants (mean age 69.5 years (IQR 60.04‐88.74), 60% women, followed for up to four decades, we identified 633 participants with new onset dementia. Moderate to severe hearing loss was associated with 30% increased risk of dementia (Alzheimer's disease HR 1.27 [95%CI 0.99‐1.62] *p* = .06; vascular dementia: HR 1.7[CI 1.06‐2.59] *p* = .027). Age stratified analysis revealed the effect was restricted to persons aged <70 years at hearing assessment (all cause dementia: HR 1.56[CI 1.19‐2.05] *p* = .001; Alzheimer's disease: HR 1.46[CI 1.07‐2.0] *p* = .017; vascular dementia: HR 2.08[CI 1.22‐3.56] *p* = .007) and strongest in *APOE‐ε4* carriers (all cause dementia (*ε*4 positiv*e*: HR 2.99 [CI 1.77‐5.06] *p* = < .0001, *ε*4 negative: HR 1.16 [CI 0.85‐1.58] *p* = 0.36); Alzheimer's disease (*ε*4 positive: HR 3.44 [CI 1.92‐6.17] *p* = < .0001, *ε*4 negative: HR 1.09 [CI 0.78‐1.54] *p* = .64)).

**Conclusion:**

Hearing loss is associated with increased risk of all cause dementia, Alzheimer's disease and vascular dementia among Framingham Study participants, with greatest risk in those aged <70 years and in *APOE‐ε4* carriers. Screening for and managing hearing loss starting in mid‐life might reduce dementia risk.